# SICyLIA-cTMT dissects redox proteome dynamics with high accuracy and depth at microgram scale

**DOI:** 10.1016/j.crmeth.2025.101210

**Published:** 2025-10-29

**Authors:** Sergio Lilla, Samuel Atkinson, Sonja Radau, Ulla-Maja Bailey, Atul Shahaji Deshmukh, Jiska van der Reest, Joanna Kirkpatrick, Thomas MacVicar, Sara Zanivan

**Affiliations:** 1 Cancer Research UK Scotland Institute, Glasgow G61 1BD, UK; 2 Institute of Medical Sciences, School of Medicine, Medical Sciences & Nutrition, University of Aberdeen, Aberdeen AB25 2ZD, UK; 3 Thermo Fisher Scientific GmbH, 63303 Dreieich, Germany; 4 Novo Nordisk Foundation Center for Basic Metabolic Research, University of Copenhagen, 2200 Copenhagen, Denmark; 5 VIB Center for Cancer Biology, 3000 Leuven, Belgium; 6 Thermo Fisher Scientific Stafford House, Boundary Way, Hemel Hempstead HP2 7GE, UK; 7 School of Cancer Sciences, University of Glasgow, Glasgow G61 1QH, UK; 8 Department of Experimental Therapeutics, University of Texas, MD Anderson Cancer Center, Houston, TX 77030, USA

**Keywords:** redox proteomics, mass spectrometry, oxidative signaling, fibroblasts, cancer, obesity, redox stress, cysteine oxidation, post-translational modification

## Abstract

Cysteine oxidative modifications are critical signaling events regulating cellular functions, but their low abundance and dynamic nature pose technical challenges. We developed the SICyLIA-TMT workflow, which sequentially labels reduced and reversibly oxidized cysteines with light and heavy iodoacetamide (IAA) within the same sample. The inclusion of tandem mass tags (TMTs) enables simultaneous quantification of oxidative modification dynamics and protein levels across multiple conditions using micrograms of material. To improve the detection of low-abundance oxidized cysteines, a dedicated TMT channel serves as a carrier for heavy IAA-labeled peptides (SICyLIA-cTMT), enhancing quantification and enabling precise stoichiometry calculations. We demonstrate the workflow’s applicability to cultured cells and full organs under stress. SICyLIA-cTMT achieves unprecedented depth and accuracy in redox proteome analysis while reducing mass spectrometry time. Combining SICyLIA-TMT with latest mass spectrometry technologies further halves the acquisition time without compromising coverage, improving throughput and enabling comprehensive studies of oxidative signaling.

## Highlights

• SICyLIA-cTMT enables simultaneous and comprehensive redox and proteome analysis
• SICyLIA-cTMT provides high sensitivity and precise stoichiometry of oxidized cysteines
• SICyLIA-cTMT works across cultured cells and whole stressed organs
• SICyLIA-cTMT achieves deep proteome coverage from micrograms of input material

## Motivation

Cysteine oxidative modifications are fundamental signaling events in regulating cellular functions under both physiological and pathological conditions. However, a major challenge in studying these modifications globally and in an unbiased manner is the low oxidation stoichiometry of most cysteine residues due to the highly reducing intracellular environment. While mass spectrometry proteomics has been crucial in advancing the field, many existing methods are labor intensive, require large sample amounts, and are biased toward specific modifications. Here, we have developed SICyLIA-carrierTMT (SICyLIA-cTMT), a simple, innovative methodology that, combined with latest mass spectrometry technologies, enables the unbiased mapping of global cysteine oxidation dynamics with exceptional depth, even from microgram-scale samples.

## Graphical abstract

**Figure:**
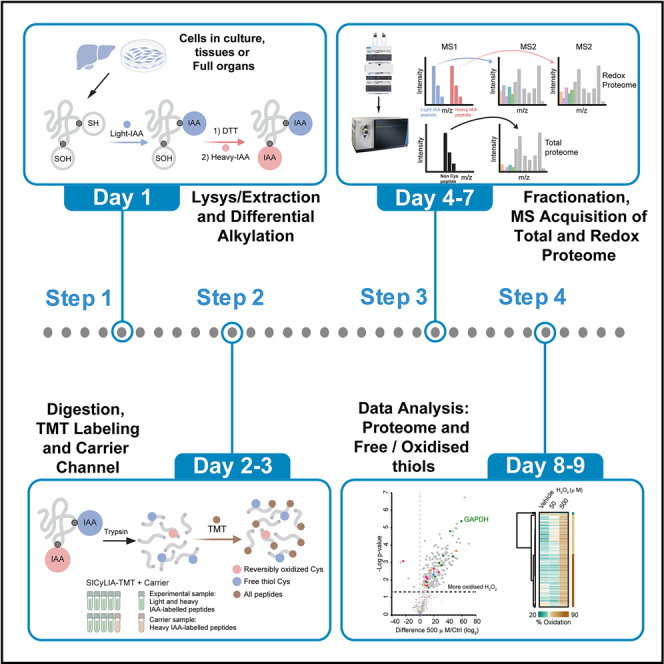

## Teaser

Cysteine oxidation controls diverse signaling pathways but remains difficult to study at scale. Lilla et al. present SICyLIA-cTMT, a quantitative mass spectrometry workflow that simultaneously measures cysteine redox dynamics and protein abundance with high accuracy from minimal input. The method enhances coverage, reduces instrument time, and enables comprehensive analysis of oxidative signaling in cells and tissues.

## Introduction

Oxidative signaling plays a pivotal role in maintaining cellular homeostasis and regulating a variety of physiological processes. Cysteine residues are particularly susceptible to oxidative modifications, such as sulfenylation, sulfinylation, and S-nitrosylation, thus playing a crucial role in oxidative signaling by modulating protein function, localization, and interactions.^1^ Most oxidative modifications are reversible, and their regulation is vital to maintain cellular health.^1^ Dysregulation of oxidative signaling can lead to oxidative stress, which is implicated in the pathogenesis of diseases, including cancer and liver dysfunctions.^2^ A deep knowledge of oxidative signaling regulation is, therefore, important to understand its roles in health and disease and enables the targeting of oxidative signaling to exploit its benefits, while mitigating its detrimental effects. Our knowledge of oxidative signaling is yet rather limited. In fact, the dynamic nature of oxidative modifications and their low-abundance due to the highly reducing intracellular environment^3^^,^^4^ have been the main challenges to overcome to advance our knowledge on oxidative signaling.

Mass spectrometry (MS) technologies coupled with redox proteomics approaches have been crucial to advance the study of oxidative signaling by enabling precise identification and accurate quantification analysis of oxidative modifications in proteins. Several multistep protocols have been developed, which use thiol reactivity agents to enrich for cysteine-containing peptides with thiols free or carrying oxidation modifications, including OxICAT,^5^ isotopic tandem orthogonal proteolysis-activity-based protein profiling,^6^ isotope-coded affinity tags (ICAT),^7^ iodoacetyl isobaric tandem mass tags^8^ or OxiTMT,^9^ and cysteine-reactive phosphate tag.^10^ Of these methods, OxICAT is based on the differential alkylation of reduced and reversibly oxidized cysteines with light and heavy ICAT reagents, followed by affinity enrichment and MS analysis to determine site-specific oxidation levels. While powerful, the method requires large sample amounts and enrichment steps, which can introduce biases or reduce throughput.

Our team has previously developed SICyLIA (stable isotope cysteine labeling with iodoacetamide), which enables measurements of cysteine oxidation dynamics at the global scale between two samples. Unlike OxICAT, SICyLIA does not require enrichment, thereby simplifying the workflow and reducing material requirements. It uses light and heavy iodoacetamide (IAA) to label reduced cysteines in each sample, and the heavy/light IAA peptide ratios serve as a direct readout of redox changes.^11^ SICyLIA provided a significant step forward in the field for its simplicity, because it does not require any enrichment step, while enabling the generation of one of the largest redox proteomes.^11^ However, SICyLIA enabled us to measure only the reduced pool of cysteine residues, and comparative analyses were limited to two experimental conditions. Furthermore, to be able to normalize modified cysteine-containing peptides by the corresponding protein amount, the proteome had to be analyzed separately, requiring long acquisition times at the MS.

To overcome these limitations, we were inspired by tandem mass tag (TMT)-based multiplexing,^12^ and by single-cell proteomics by MS (SCoPE-MS).^13^ TMTs are widely used isobaric chemical labels that allow for the simultaneous quantification of peptides from multiple samples (up to 35) in a single MS run, improving throughput and reproducibility.^12^ SCoPE-MS is a method developed for single-cell proteomics that introduced the use of a carrier channel, a pooled sample in one TMT channel, to increase peptide signal intensity and improve the detection of low-abundance peptides during MS analysis, as it provides enough ions to trigger tandem MS fragmentation.

Building on these advances, we developed SICyLIA-cTMT, which integrates differential labeling of reduced and reversibly oxidized cysteine residues with TMT labeling and a carrier TMT channel to boost detection of oxidation signals. This hybrid approach merges the simplicity and enrichment-free nature of SICyLIA with the sensitivity and multiplexing capability of TMT-based workflows. SICyLIA-cTMT allows for accurate quantification of oxidative dynamics of thousands of cysteine residues and total proteomes across multiple biological conditions, starting from only micrograms of sample.

## Results

### SICyLIA-TMT enables direct measurement of reversibly oxidized cysteine residues

**Figure 1:**
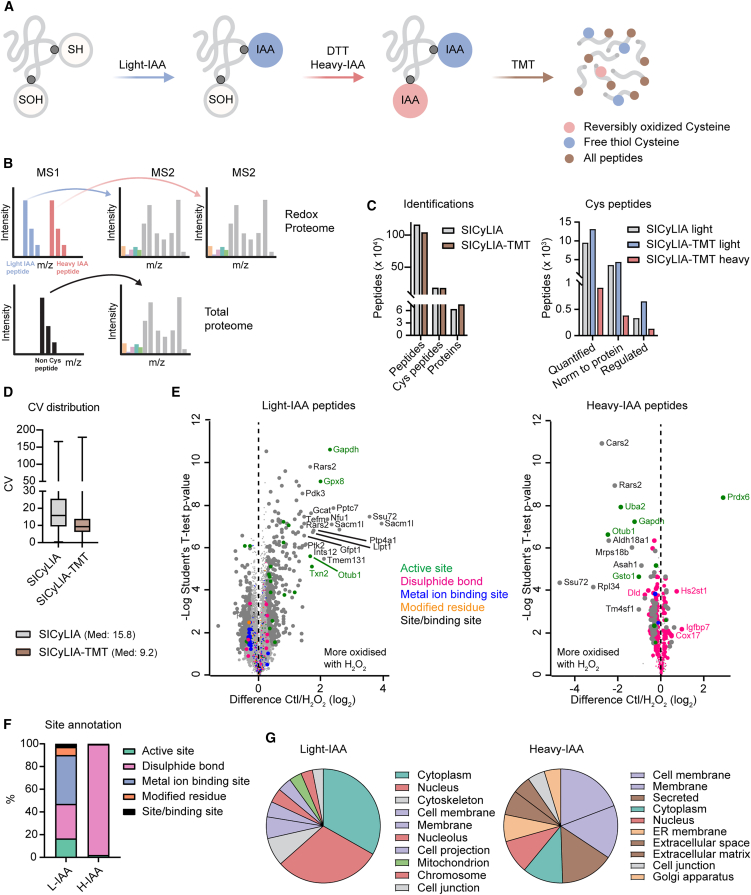
SICyLIA-TMT is a simple workflow for high-precision measurement of oxidative signaling (A) Schematic workflow of the SICyLIA-TMT labeling workflow. (B) Schematic MS spectrum of a non-cysteine peptide (bottom) and cysteine-containing peptide pair labeled with light (blue, reduced) and heavy (red, reversibly oxidized) IAA (top). Corresponding tandem MS spectra highlight TMT reporter ions enabling quantification across conditions. (C) Number of cysteine peptides and proteins identified in lysate of murine kidney cells treated with hydrogen peroxide (H_2_O_2_) or vehicle using SICyLIA or SICyLIA-TMT workflows. (D) Boxplot (min to max) showing CV for peptides from murine kidney cells analyzed using SICyLIA^11^ or SICyLIA-TMT workflows (n_SICyLIA_ = 11,224 peptides; n_SICyLIA-TMT_ = 14,020 peptides). (E) Volcano plots showing differences in oxidative modification levels in cysteine peptides in H_2_O_2_-treated kidney cells. Larger dots = cysteine significantly regulated (t test, 5% FDR). Site annotation based on UniProt. (F) Proportion of IAA-labeled cysteine residues annotated to the indicated categories (UniProt as in E) quantified with SICyLIA-TMT workflow in kidney cells. (G) Pie charts showing top 10 Gene Ontology Cellular Component categories of proteins with IAA-labeled cysteines, based on category counting Perseus analysis.

In a typical SICyLIA-TMT workflow, proteins are extracted from a biological sample using light IAA-containing lysis buffer to irreversibly alkylate free thiol groups on cysteine residues. Cysteine residues carrying reversible oxidative modifications are then reduced with a reducing agent such as DTT, and the newly generated thiol groups are irreversibly alkylated with heavy IAA (Figure 1A). Proteins are then digested, and peptides are labeled with TMT (Figure 1A) and mixed and fractionated with offline high pH reverse-phase chromatography before analysis on a high-resolution MS instrument (Figure 1B). Using MaxQuant software for MS data analysis and Perseus software for downstream analysis, SICyLIA-TMT enables to measure both protein abundance, using non-IAA labeled peptides, and oxidation status of cysteine residues, using IAA-labeled peptides, across as many samples as the labels of the multiplex TMT used for the labeling (Figure 1B and STAR Methods). The fact that we can measure the proteome within the same sample is important 2-fold: to normalize oxidation levels of each cysteine residue for the corresponding protein abundance, which enables precise quantification of oxidation changes across experimental conditions, and to measure in-depth proteome changes across experimental conditions. In SICyLIA, this step was sub-optimal because samples to measure the proteomes had to be processed and measured separately at the MS, because of the need of further labeling of the alkylated peptides with dimethyl reagents.^11^

To assess the performance of the SICyLIA-TMT workflow, we benchmarked it against SICyLIA^11^ using freshly prepared samples from the same biological model of acute oxidative stress signaling triggered by 500 μM H_2_O_2_ 15-min stimulation in murine kidney cells used for SICyLIA. However, while with SICyLIA we used 150 μg of lysate for each sample, SICyLIA-TMT enabled us to use only 50 μg per sample. Another major difference was the measuring time at the mass spectrometer, a Q-Exactive HF for both studies, which TMT reduced to one-third of that used with SICyLIA, from 6.2 days to 1.6 days. Despite this large difference, the two methods identified similar numbers of peptides and cysteine-containing peptides, as well as proteins (Figure 1C and Table S1). Moreover, SICyLIA-TMT enabled accurate quantification (peptide levels normalized to the corresponding protein levels) of 48% more IAA-labeled peptides, of which 13,109 contained reduced cysteine residues (light IAA) and 911 contained reversibly oxidized cysteine residues (heavy IAA) (Figure 1C). The difference between heavy IAA- and light IAA-labeled peptides is consistent with the highly reducing intracellular environment.^3^^,^^4^ Conversely, SICyLIA quantified 9,479 peptides with reduced (light IAM) cysteine residues (Figure 1C). With SICyLIA-TMT we also observed a pronounced reduction in the median coefficient of variation (CV) of the quantified peptides across biological replicates (median CV < 10%), almost 2-fold lower than in the original SICyLIA workflow (Figure 1D). While TMT ratio compression can, in principle, artifactually dampen variability, the magnitude of the improvement seen here suggests that multiplexing-driven noise reduction is a major contributor; nonetheless, we cannot exclude that ratio compression accounts for part of the effect. The higher reproducibility enabled more stringent statistical testing. Using Student’s t test with 1% permutation-based false discovery rate (FDR) identified 782 sites significantly regulated, of which 651 were light IAA labeled and 131 were heavy IAA labeled (Figures 1C and 1E and Table S1). These were more than 2-fold of those identified with SICyLIA.^11^ These results highlight the higher sensitivity and improved quantitative reproducibility of the SICyLIA-TMT workflow and suggest that the response to H_2_O_2_ is more complex than we previously reported. Overall, results corroborated our previous SICyLIA data indicating that cells were actively detoxifying reactive oxygen species. Light IAA-labeled cysteine residues included known protein regulatory sites (e.g., active sites, disulfide, metal binding) significantly regulated (Figure 1E and Table S1), such as the catalytic cysteine 150 of glyceraldehyde-3-phosphate dehydrogenase (Gapdh), which has been previously shown to carry oxidative modifications^14^ and whose activity we demonstrated to be regulated by H_2_O_2_. Conversely, heavy IAA labeling unraveled that the majority of the reversibly oxidized cysteine residues captured with SICyLIA-TMT are involved in disulfide bonds (Figures 1E and 1F). However, there were also few active sites, including the catalytic center of the peroxidase activity cysteine 47 of Prdx6,^15^ which is a member of the peroxiredoxin family, which are well-characterized antioxidant enzymes that control peroxide levels in the cell. Notably, for a subset of the heavy IAA-regulated sites, both light and heavy IAA-labeled counterparts were quantified. Among these, 10 sites were also regulated in the light IAA-labeled form, with 9 showing opposite regulation (e.g., Cys150 of GAPDH, Cys88 of ALDH18A1; see Table S1), providing direct proof that changes in reduced levels were due to increased oxidation. Finally, subcellular localization annotation using location categories in UniProt, showed that light IAA-labeled cysteine residues are mainly found in proteins annotated as cytoplasmic and nuclear, while heavy IAA-labeled ones are associated with proteins located at the cell membrane and extracellular space (Figure 1G). This result is consistent with disulfide bonds forming during the biosynthesis of secreted proteins in the endoplasmic reticulum (ER) and with disulfide bonds being unstable in the reducing environment of most cellular compartments, while being stable in the ER.^16^ SICyLIA-TMT was also superior at the proteome level. With SICyLIA-TMT, we identified 7,486 proteins, compared to 6,356 with SICyLIA (Figure 1C). Moreover, SICyLIA quantified 2,920 proteins (with heavy/light ratio) across all replicate experiments, while TMT enabled the quantification of all the identified proteins, 7,486, across all samples (Table S1). However, the increased proteome coverage with SICyLIA-TMT was likely largely attributable to the enhanced multiplexing and quantification capabilities of TMT.

In summary, SICyLIA-TMT enables global measurements of oxidative signaling dynamics and has superior performances compared to SICyLIA for depth and precision of IAA-labeled peptide quantification. Furthermore, it significantly reduced analysis time, while retaining its simplicity.

### SICyLIA-TMT coupled with a heavy IAA carrier enables in-depth measurement of oxidative modification stoichiometry of cysteine residues in cells

**Figure 2:**
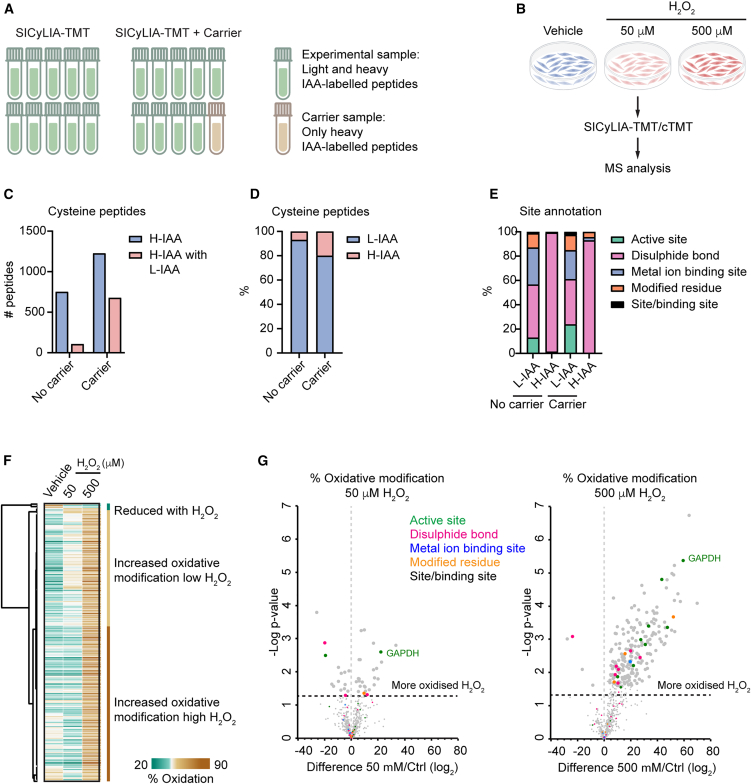
A heavy IAA-labeled carrier enhances quantification of reversibly oxidized cysteines under oxidative stress (A) Experimental design of SICyLIA-TMT (left) and SICyLIA-cTMT with carrier (right). (B) Schematic representation of CAFs treated with vehicle or increasing concentrations of H_2_O_2_ for 15 min and analyzed with SICyLIA-TMT ± carrier. (C) Bar plot showing number of total heavy IAA-labeled peptides (H-IAA) identified in CAFs using SICyLIA-TMT ± carrier and those with a quantified light IAA (L-IAA) counterpart. (D) Bar plot showing the percentage of IAA-labeled peptides identified in CAFs using SICyLIA-TMT ± carrier channel. (E) Site annotation (based on UniProt database) of cysteine peptides from CAFs quantified with SICyLIA-TMT ± carrier. (F) Heatmap and clustering (Pearson correlation) of peptides with significantly regulated oxidation levels (ANOVA, FDR <5%). (G) Volcano plots of cysteine oxidation stoichiometry under low- or high-dose H_2_O_2_ stimulation in CAFs. Horizontal line indicates *p*-value of 0.05. Larger dots are peptides with *p* value ≤0.05 (low H_2_O_2_) or that passed t test with FDR <5% (high H_2_O_2_). Site annotations based on UniProt database.

The extent of the change in thiol oxidation stoichiometry could pinpoint changes that could significantly alter protein activity and, therefore, be biologically significant. SICyLIA-TMT enables the assessment of thiol oxidation stoichiometry. In fact, when both light IAA and heavy IAA forms of the same cysteine-containing peptide are quantified, and we assume that their sum corresponds to 100% of that specific cysteine peptide, we can estimate the thiol oxidation stoichiometry as the proportion of the peptide that carries a reversible oxidative modification (see STAR Methods). However, due to low abundance of the heavy IAA-labeled peptides, we could quantify only a few such peptides (59, Table S1). To boost sensitivity for heavy IAA peptides, we used one of the TMT channels for a carrier proteome in which all the cysteine residues had been first reduced and then labeled with heavy IAA. To assess the performance of the carrier, we measured oxidative signaling triggered by H_2_O_2_ stimulation in a line of previously characterized human mammary breast cancer-associated fibroblasts (CAFs)^17^^,^^18^ using SICyLIA-TMT without or with carrier (SICyLIA-cTMT) (see STAR Methods). As carrier, we used heavy IAA-labeled peptides derived from a lysate of untreated CAFs, which was fully reduced with DTT prior to heavy IAA labeling (Figure 2A). Moreover, to assess the suitability of this approach to measure different strengths of signaling, we measured the redox proteome of CAFs treated with vehicle, or with H_2_O_2_ at a concentration of 500 μM (as for the murine kidney cells, Figure 1) or 50 μM (Figure 2B), which is in a range that has been shown to have limited cytotoxicity in many cell types.^19^

Overall, the use of the carrier led to a sizable increase in identified heavy IAA-labeled peptides, 1,255 compared to 751 without the carrier (Figure 2C and Tables S2 and S3), which also represented a higher percentage of peptides labeled with IAA (21% compared to 7% without the carrier, Figure 2D). Strikingly, the number of peptides for which both heavy IAA- and light IAA-labeled peptides were quantified improved by almost 7-fold (677 with the carrier and only 107 without the carrier, Figure 2C and Tables S2 and S3). Next, we annotated the light IAA- and heavy IAA-identified sites and, similarly to the murine kidney cells, we found a variety of functions associated with the light IAA sites, while most of the heavy-labeled, reversibly oxidized, cysteine residues were associated with disulfide bonds, again reflecting the inherent chemical and structural bias of our method and the influence of limited coverage toward this abundant class of modifications (Figure 2E). However, the improved depth reached with the carrier enabled the identification of sites involved in metal ion binding and modified sites also among the heavy IAA-labeled sites (Figure 2E).

Next, we focused on the 677 peptides for which we could calculate the stoichiometry and performed an ANOVA test (FDR<5%) to identify sites significantly regulated by hydrogen peroxide. With the carrier we identified 195 significantly regulated cysteine residues (Figure 2F and Table S3), while we identified only 47 without the carrier (Table S2). Most of the regulated sites increased oxidative modification levels upon H_2_O_2_ treatment and reached statistical significance upon high dose of H_2_O_2_ treatment (Figure 2G). However, a trend toward increased oxidation was observed already for a subset of cysteine residues at low-dose hydrogen peroxide (Figures 2F and 2G), including Cys 152 of GAPDH, which, as also shown above (Cys 150 of murine Gapdh), is known to respond to redox stress (Figure 2G).

Hence, using a carrier channel in SICyLIA-TMT dramatically improved the sensitivity for low-abundance peptides and the depth of oxidative stoichiometry measurements for a more profound understanding of oxidation modification dynamics.

### SICyLIA-cTMT enables in-depth measurement of oxidative modification stoichiometry of cysteine residues in tissue samples

Next, we assessed the performance of SICyLIA-TMT with and without a carrier applied to a whole organ in a model relevant for oxidative signaling. We selected the liver as it is a central metabolic organ with high oxidative capacity and a major site of redox regulation, making it particularly suitable for evaluating redox-sensitive proteomic workflows. We used a well-characterized and widely used model of obesity, the *ob/ob* mouse.^20^ This mouse carries a mutation for the gene encoding the hormone leptin,^21^ which modulates feeding behaviors by signaling to the hypothalamus.^22^ These mice develop obesity, insulin resistance, and hepatic steatosis.^23^^,^^24^ Previous MS proteomic analysis of the liver of these mice showed extensive changes related to metabolism, including increased proliferation of peroxisomes,^25^ which play major roles in H_2_O_2_ metabolism.^26^

**Figure 3:**
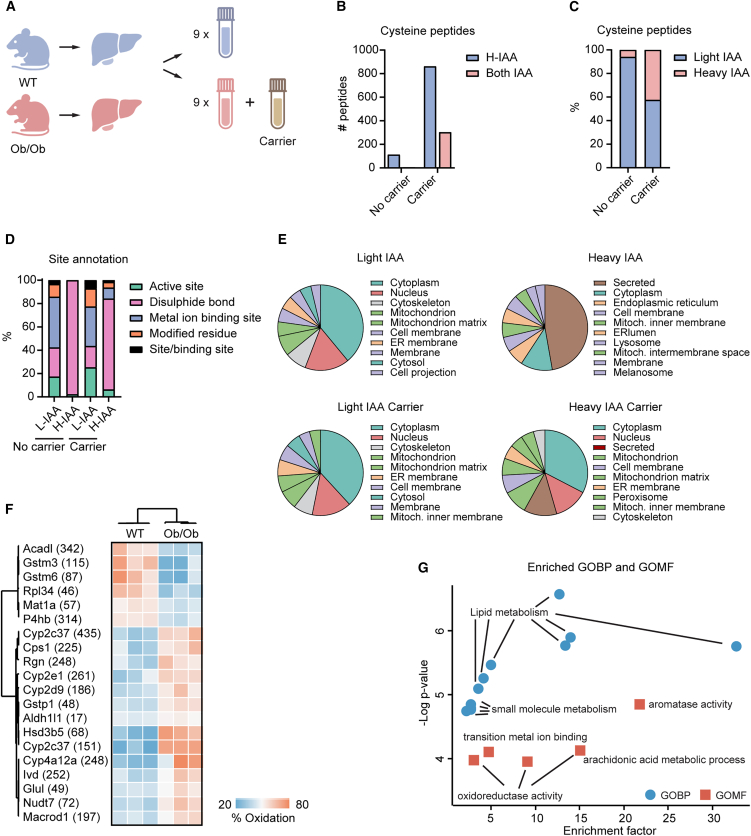
SICyLIA-cTMT improves quantification of reversibly oxidized cysteines in metabolically stressed livers (A) Schematic of WT and *Ob/Ob* liver tissue samples analyzed with SICyLIA-TMT or SICyLIA-cTMT. (B) Bar plot of heavy IAA-labeled (H-IAA) peptides identified in liver samples using SICyLIA-TMT ± carrier channel and those with a light IAA-labeled (L-IAA) counterpart. (C) Bar plot showing the percentage of IAA-labeled peptides identified in liver tissue using SICyLIA-TMT ± carrier. (D) Site annotation (UniProt database) of liver tissue peptides quantified with SICyLIA-TMT ± carrier. (E) Pie charts of top 10 Gene Ontology Cellular Component categories to which oxidized liver proteins were annotated based on category counting Perseus analysis. (F) Heatmap and clustering (Person correlation) of cysteine peptides with significantly regulated stoichiometry between WT and *Ob/Ob* livers (ANOVA; FDR <5%). (G) Dot plots of Gene Ontology Molecular Function (GOMF) and Biological Processes (GOBP) significantly enriched (Fisher test; FDR <0.02) among regulated sites in (F). “Relative enrichment” by gene names (Perseus analysis).

We measured the redox proteome of liver samples from wild-type (WT) or *Ob/Ob* mice with the SICyLIA-TMT or SICyLIA-cTMT workflow (Figure 3A and STAR Methods). The carrier strikingly increased the depth of the reversibly oxidized sites. In fact, with the carrier we quantified 862 heavy IAA-labeled peptides, while only 113 without (Figure 3B and Tables S4 and S5), which corresponded to 35% and 5% of the IAA-labeled peptides, respectively (Figure 3C). Like the experiment with the CAFs, functional annotation of the IAA-labeled cysteine sites showed that the carrier enabled to increase the variety of the heavy-labeled, reversibly oxidized sites. In fact, in addition to the multitude of sites involved in disulfide bonds, the quantified sites included binding sites, which were not found with the normal SICyLIA-TMT workflow (Figure 3D and Tables S4 and S5). To understand further the impact of SICyLIA-cTMT on the measured redox proteome, we looked at the subcellular location of the proteins carrying IAA-labeled cysteine residues. As for the kidney cells, the pool of light IAA-labeled peptides quantified with SICyLIA-TMT contained mostly cytosolic and nuclear proteins, while the pool of heavy IAA-labeled peptides contained mostly secreted proteins (Figure 3E and Tables S4 and S5). Additionally, both pools contained mitochondrial proteins, particularly those localizing in the inner mitochondrial membrane when using the carrier, consistent with disulfide bond relays being important in mitochondrial protein import in intermembrane space.^27^^,^^28^ Notably, using the carrier enabled to quantify the oxidation status of a large subset of cytosolic and nuclear proteins in addition to the secreted ones (Figure 3E and Table S5).

Finally, as in the CAF analysis, using the carrier dramatically enhanced the quantification of peptides labeled with both heavy IAA and light IAA, which were only 6 for the samples without carrier, while reached over 300 peptides for those processed with SICyLIA-cTMT (Figure 3B and Tables S4 and S5).

Of the 300 cysteine-containing peptides, 20 had significantly different oxidation status between WT and *Ob/Ob* mice, of which 14 were more oxidized and 6 were more reduced in the liver of *Ob/Ob* mice (Figure 3F and Table S5). Proteins with regulated oxidative modifications were significantly enriched for proteins involved in oxidoreductase activity, and in metabolic processes, including lipid and small molecule metabolism (Figure 3G and Table S5). Notably, the list included several P450 enzymes, a family of enzymes involved in drug metabolism, hormone regulation, and detoxification. Of particular interest is the cysteine 61 of the S-adenosylmethionine synthase isoform type-1 (Mat1a), which can potentially impact the function of the protein, with evidence in the rat homolog protein showing it to form a disulfide bond with cysteine 35.^29^

SICyLIA-cTMT dramatically improved the depth of oxidative stoichiometry measurements at the full organ level and pinpointed altered oxidative modifications in the metabolically challenged liver of obese mice.

### Orbitrap Astral mass spectrometer boosts depth and throughput of redox proteomes measured with SICyLIA-TMT workflow

**Figure 4:**
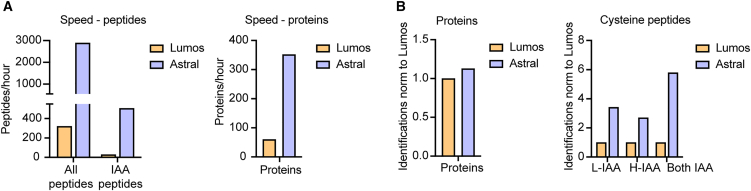
Orbitrap Astral increases depth and speed of the oxidative signaling measured with SICyLIA-TMT (A) Bar plots showing total proteins, peptides, and IAA-labeled peptides identified per hour in liver extracts labeled with SICyLIA-TMT using Orbitrap Lumos or Orbitrap Astral instrument. (B) Bar plots showing number of liver proteins and cysteine peptides labeled with light (L) or heavy (H) IAA or both using SICyLIA-TMT identified using Orbitrap Lumos or Orbitrap Astral instrument.

Increased speed and sensitivity of latest MS instruments have dramatically improved the proteome depth that can be achieved and throughput. We assessed the impact of integrating latest technologies in our SICyLIA-TMT workflow and analyzed the same CAF and liver lysates described above in an Orbitrap Astral mass spectrometer.^30^ For this comparative analysis, we used TMT10Plex, and after fractionation, the TMT-labeled peptides were pooled into 7 fractions, which were run in an Orbitrap Lumos and in an Orbitrap Astral. The first major difference was the overall measuring time at the mass spectrometer, which was almost 3 days on the Orbitrap Lumos and just over half a day on the Orbitrap Astral. As expected, the Orbitrap Astral was much faster and identified higher number of both peptides and proteins per hour than the Orbitrap Lumos (Figure 4A and Table S6). Such difference in speed between instruments led to identify 3- to 6-fold more peptides and IAA-labeled peptides in the Astral than in the Lumos despite the large difference in measuring time (Figure 4B). We also found a large increase in the number of cysteine residues for which both light and heavy counterparts were quantified to calculate the percentage of oxidation (Figure 4B). Conversely, the number of proteins identified was similar in the two instruments (Figure 4B and Table S6). A recent study has proposed optimized signal-to-noise and resolution-based filtering strategies that improve TMT quantification reproducibility on the Orbitrap Astral and can serve as a guideline for future experiments.^31^ Hence, combining SICyLIA-TMT with the latest MS technologies can further reduce measuring time in the mass spectrometer while enabling the measurement of oxidative signaling dynamics with greater depth.

## Discussion

In this study, we developed and validated SICyLIA-cTMT, a powerful workflow for the comprehensive and accurate measurement of cysteine oxidation dynamics. By combining differential IAA labeling with TMT multiplexing and a carrier channel, SICyLIA-cTMT improves sensitivity, throughput, and sample efficiency compared to previous redox proteomics methods.^8^^,^^9^^,^^10^^,^^32^

The carrier channel substantially increased the detection of heavy IAA-labeled peptides and allowed stoichiometry calculations for redox-sensitive cysteines. This revealed oxidation events not captured by earlier approaches and improved quantitative reproducibility, likely due to simultaneous multiplexed analysis. Importantly, SICyLIA-cTMT also improves cost-effectiveness and scalability. As detailed in Table S7, the workflow halved the cost per experimental sample and reduced processing time from 5.6 to 4 days. Crucially, it enabled the analysis of up to 18 experimental samples in parallel, compared to only 4 (2 in duplicate) with SICyLIA. When combined with an Orbitrap Astral mass spectrometer, analysis time was further reduced to less than 2 days, while still delivering greater depth of cysteine oxidation quantification. These improvements underscore the practicality of SICyLIA-cTMT for large-scale studies.

SICyLIA-TMT identified over 13,000 light IAA-labeled and over 900 heavy IAA-labeled peptides, surpassing the capacity of SICyLIA. Improved reproducibility across replicates, likely due to simultaneous analysis of all experimental conditions via TMT, further underscores the method’s robustness. A similar observation comes from a recent work in which cysteine labeling and TMT have been combined to measure proteome and reduced cysteine levels.^33^

Applied to oxidative stress in murine kidney cells, the method uncovered more than 700 regulated cysteine residues, many at disulfide bonds or active sites, and enabled stoichiometry estimates for key redox switches such as GAPDH and PRDX6.^14^ Subcellular localization analysis revealed compartment-specific redox responses consistent with known oxidative environments,^16^ and improved detection of cytosolic and nuclear oxidized cysteines showed how the carrier strategy reduces previous detection biases.

Extending the workflow to obese mouse liver extracts demonstrated versatility in complex tissues. The carrier-enabled workflow revealed oxidation changes in metabolic enzymes pointing to a link between redox imbalance and metabolic dysfunction in obesity. These findings are in line with previous studies showing lipid metabolism-related proteome to be highly dysregulated in the liver of obese mice^25^ and suggest additional avenues for investigating the functional consequences of redox modifications in this context.

In summary, SICyLIA-cTMT provides a sensitive, reproducible, and scalable approach for quantifying cysteine oxidation in diverse systems, offering a valuable tool for dissecting redox regulation in health and disease.

### Limitations of the study

SICyLIA-cTMT can be applied to any type of biological sample; however, it requires rapid alkylation of reduced cysteines (e.g., by including IAA in lysis buffer) to prevent artifactual oxidation during sample preparation. Moreover, SICyLIA-cTMT does not capture irreversible cysteine oxidations (e.g., sulfinic or sulfonic acids), which may lead to underestimation of total oxidation. Also, it does not distinguish among different reversible oxidative modifications. To characterize specific modifications, enrichment or targeted chemistries are needed. Finally, because the workflow avoids enrichment, coverage of oxidized peptides is lower than in enrichment-based approaches. However, this trade-off enables analysis from low-input samples and reduces bias.

## Resource availability

### Lead contact

Requests for additional information, resources, or reagents should be directed to the lead contact, Sara Zanivan (srzanivan@mdanderson.org), who will provide the requested materials and address any inquiries.

### Materials availability

The study did not generate or use any unique materials.

### Data and code availability

• The raw MS files and search/identification files obtained with MaxQuant have been deposited to the ProteomeXchange via the PRIDE partner repository with dataset identifiers PRIDE: PXD061795, PRIDE: PXD061806, PRIDE: PXD061827, PRIDE: PXD061848, PRIDE: PXD061855, and PRIDE: PXD061865.
• This study did not generate or report any original code.
• Additional details necessary to reanalyze the data presented in this study can be obtained from the lead contact upon request.

## Acknowledgments

We would like to thank Dr. Marie Bjornholm for her assistance with the rodent experiments and for kindly providing liver tissue samples, Dr. Eyal Gottlieb for providing murine kidney cells, and the PRIDE team. We thank the Cancer Research UK (CRUK) Scotland Institute core research services and the proteomics advanced technology facility. This work was funded by CRUK core funding to the CRUK Scotland Institute (grant no. A31287) and CRUK Glasgow Centre (grant no. A18076). S.Z. was funded by Stand Up To Cancer campaign for CRUK (grant no. A29800 to S.Z.) and by research funds from The 10.13039/100007313University of Texas MD Anderson Cancer Center. U.-M.B. is funded by 10.13039/501100000289CRUK (grant no. EDDPJT-Nov21\100006 to S.Z.). T.M. was funded by CRUK Career Development Fellowship (RCCFELCDF-May21\100001). A.S.D was funded by 10.13039/501100009708Novo Nordisk Foundation (grant no. NNF18CC0034900). Some schematics were created with BioRender.

## Author contributions

Conceptualization, S.Z. and S.L.; methodology, S.Z., S.L., S.R., and J.K.; investigation, S.L., S.A., S.R., and J.v.d.R.; writing – original draft, S.Z., S.L., and U.-M.B.; writing – review & editing, S.Z.; visualization, S.Z. and S.L.; supervision, S.Z.; resources, U.-M.B., J.K., A.S.D., and T.M.; funding acquisition, S.Z.

## Declaration of interests

J.K. and S.R. are both employees of Thermo Fisher Scientific, the manufacturer of the Orbitrap Astral mass spectrometer used in this study.

## Declaration of generative AI and AI-assisted technologies in the writing process

During the preparation of this work, the authors used ChatGPT to improve the writing style of the introduction and discussion. After using this tool or service, the authors reviewed and edited the content as needed and take full responsibility for the content of the publication.

## STAR★Methods

### Key resources table

**Table:** 

REAGENT or RESOURCE	SOURCE	IDENTIFIER
**Biological samples**		

Liver tissue wild type mice	Dr. Atul Deshmukh	N/A
Liver tissue from obese mice	Dr. Atul Deshmukh	N/A

**Chemicals, peptides, and recombinant proteins**		

Water LC/MS, LiChrosolv®	Supelco	Cat# 1.15333
20% Sodium dodecyl sulfate (SDS)	Fisher Scientific	Cat# 10607443
Tris-HCl, 1M Solution	Thermo Fisher Scientific	Cat# J22638.AP
Iodoacetamide (IAA)	Sigma-Aldrich	Cat# I1149
Iodoacetamide heavy (I^13^CD_2_^13^CONH_2_)	Sigma-Aldrich	Cat# 721328
DL-Dithiothreitol	Sigma-Aldrich	Cat# 43819
HEPES (1M solution)	Gibco Fisher scientific	Cat# 11560496
Acetone HPLC Plus	Sigma-Aldrich	Cat# 650501
Endoproteinase Lys-C	Biolabs	Cat# P8109S
Trypsin Gold, mass spectrometry grade	Promega	Cat# V5280
Acetonitrile ≥99.9%, HiPerSolv CHROMANORM®	VWR Chemicals	Cat# 20060.320
TMTpro 16-plex, Label Reagent Set	Thermo Fisher Scientific	Cat# A44522
TMTpro 18-plex, TMTpro-134C and 135N Label Reagents	Thermo Fisher Scientific	Cat# A52047
Hydroxylamine solution 50 wt. % in H_2_O, 99.999%	Sigma-Aldrich	Cat# 467804
Trifluoroacetic acid	Sigma-Aldrich	Cat# 302031
Formic acid 98–100% EMSURE®	Supelco	Cat# 1.00264
Ammonia solution 25%	Supelco	Cat# 5.33003
Kinetex EVO - 5 μm, 100 Å - 150 × 2.1 mm i.d.	Phenomenex	Cat# 00F-4633-AN
Acetic acid glacial 99.8–100.0%, ARISTAR®	VWR Chemicals	Cat# 450013Y
Sep-Pak C18 1 cc Vac Cartridge, 50 mg Sorbent	Waters	Cat# WAT054955
ReproSil-Pur C18-AQ, 1.9 μm resin	Dr. Maisch GmbH	Cat# r119.aq.
Pierce™ BSA Protein Digest, MS grade	Thermo Fisher Scientific	Cat# 88341
Pierce™ HeLa Protein Digest Standard	Thermo Fisher Scientific	Cat# 88328

**Critical commercial assays**		

Pierce™ BCA Protein Assay Kit	Thermo Fisher Scientific	Cat# 23227
Precellys® Lysing kit	Bertin Instruments	Cat# KT03961-1-203.05

**Deposited data**		

part1 = Murine kidney cells upon hydrogen peroxide treatment.	PRIDE Archive	PXD061795
part2 = Human mammary breast cancer associated fibroblasts with carrier channel.	PRIDE Archive	PXD061806
part3 = Metabolically stressed liver tissues.	PRIDE Archive	PXD061827
part4 = Metabolically stressed liver tissues Orbitrap ASTRAL.	PRIDE Archive	PXD061848
part5 = Human mammary breast cancer associated fibroblasts.	PRIDE Archive	PXD061855
part6 = Metabolically stressed liver tissues without carrier.	PRIDE Archive	PXD061865

**Experimental models: Cell lines**		

Murine kidney epithelial cells	Dr. Eyal Gottlieb	N/A
Human mammary cancer associated fibroblasts	Dr. Akira Orimo	N/A

**Experimental models: Organisms/strains**		

Wild-type lean controls male mice on a C57BL/6J background	Charles River Laboratories	N/A
Four-month-old male *ob/ob* mice on a C57BL/6J background	Charles River Laboratories	N/A

**Software and algorithms**		

MaxQuant 2.3.0.0	N/A	https://www.maxquant.org/
Perseus 1.6.15.0	N/A	https://maxquant.net/perseus/
UniProt database human	UniProtKB	https://www.uniprot.org/proteomes/UP000005640
UniProt database mouse	UniProtKB	https://www.uniprot.org/proteomes/UP000000589

**Other**		

Orbitrap Fusion™ Lumos™ Tribrid™ Mass Spectrometer	Thermo Fisher Scientific	Cat# IQLAAEGAAPFADBMBHQ
Easy-nLC 1200 LC System	Thermo Fisher Scientific	Cat# LC140
50 cm Emitters	CoAnn Technologies	Cat# ICT36007508-50
ABIRD - The Active Background Ion Reduction Device for nano spray LC/MS	ESI Solutions	Cat# ABIRD
SpeedVac vacuum concentrator	Thermo Fisher Scientific	Cat# RVT5105
Eppendorf Thermomixer or equivalent	Eppendorf	Cat# 5355
Eppendorf microcentrifuge or equivalent	Eppendorf	Cat# 5430
Agilent 1260 Infinity HPLC with Fraction collector and Open Lab CDS software	Agilent	Cat# G1311A
Cell scraper	Various suppliers	N/A
Incubator for cells	Various suppliers	N/A
Biological safety cabinet for cell culture	Various suppliers	N/A
Chemical safety cabinet	Various suppliers	N/A
Precellys24	Bertin Instruments	Cat# P002391-P24T0-A.0

### Experimental model and study participant details

#### Cancer associated fibroblasts stimulation and lysis

Human mammary cancer associated fibroblasts (CAFs) were kindly provided by Dr. Akira Orimo.^17^ CAFs were cultured in DMEM (Gibco) supplemented with 10% FBS (Gibco) and 1% penicillin-streptomycin (Gibco) and seeded at 1 x 10^6^ cells in 10 cm plate for 24 h. After PBS wash, cells were treated with 50 or 500 μM H_2_O_2_ or vehicle for 15 min and immediately lysed.

#### Murine kidney cells

Murine *Fh*^*fl/fl*^ cells were isolated, immortalised, and authenticated as described previously.^34^ Cells were grown in DMEM supplemented with 2 mM glutamine and 10% FBS. Cells were treated with 500 μM hydrogen peroxide 15 min prior to protein extraction.

#### Liver tissue preparation

All animal experiments were performed at the Karolinska institute, Stockholm, Sweden and were approved by the Regional Animal Ethical Committee (Stockholm, Sweden). Four-month-old male *ob/ob* mice^21^ and wild-type (WT) lean controls on a C57BL/6J background were obtained from Charles River (Italy). For this study only male mice were used, therefore conclusions cannot be generalized and remain limited to males. The mice had *ad libitum* access to water and standard rodent chow and were housed in a temperature- (22°C–24°C) and light-controlled (12 h light/dark cycle) environment. For liver proteome analysis, mice were fasted for 4 h before being anesthetized with Avertin (2,2,2-tribromoethanol 99% and tertiary amyl alcohol (1:1 w/v), 500 mg⋅kg−1 body weight). Livers were then excised, snap-frozen in liquid nitrogen, and stored at −80°C until further processing.

### Method details

#### Cancer associated fibroblasts lysis

Cells were lysed with 100 μL of lysis buffer, 100 mM Tris-HCl pH 7.4, 2% SDS, 55 mM light IAA. Light IAA was used for irreversible alkylation (formation of carbamidomethyl groups) of free thiol groups on cysteine residues. Samples were then incubated at room temperature in the dark, sonicated, and centrifuged at 16,000 g for 30 min. Supernatants were then incubated for 1 h at room temperature with mixing in the dark to enable full labeling of cysteine residues with IAA. Lysates were subsequently processed with SICyLIA-TMT workflow.CAFs used for the carrier samples were lysed as described above but using lysis buffer without light IAA.

#### Liver protein extraction

Frozen liver tissue from 3 male *ob/ob* mice and 3 male wild type (WT) mice lean controls on a C57BL/6J background^25^ were weighed and extracted in 100 mM HEPES buffer pH 7.4, 4% SDS with 55 mM light IAA using a bead-based homogenizer Precellys24 (Bertin Instruments) under dry ice vapor at 5,500 rpm, three times for 30 s. Extracts were sonicated on ice 4 times for 5 s with amplitude set at 30 (Fisherbrand). Samples were incubated in the dark for 1 h at 25°C mixing at 1,200 rpm to allow alkylation of thiol groups on cysteine residues with light IAA. Samples were then centrifuged at 16,000 g at 4°C for 10 min and the supernatants were transferred to clean tubes. Extracts were stored at −80°C until further processing with SICyLIA-TMT workflow.

#### SICyLIA-TMT workflow

Protein concentration was determined using a BCA Protein Assay Kit (Pierce). Proteins were reduced with DTT, final concentration 10 mM, to reduce reversible oxidative modifications on cysteine residues, for 1 h at room temperature, shaking 1,000 rpm, which were subsequently alkylated in the dark with heavy IAA (^13^C_2_D_2_H_2_INO, Sigma), 55 mM final concentration, 1h at room temperature, shaking 1,000 rpm. Alkylated proteins were precipitated in two steps using 24% and 10% solutions of trichloroacetic acid (TCA). In both steps, pellets were incubated at 4°C for 10 min and centrifuged at 18,000 g for 5 min. Supernatants were carefully aspirated and pellets were finally washed with water until the supernatant reached neutral pH. For CAFs treated with hydrogen peroxide, proteins were precipitated with ice-cold acetone overnight at −20°C. Precipitated proteins were washed twice with ice-cold acetone. Washed pellets were reconstituted in HEPES 200 mM and digested first with Endoproteinase Lys-C (Wako chemicals) for one hour, followed by Trypsin overnight (Promega). The digested peptides from each experiment, pool or carrier samples, were differentially labeled using Thermo Fisher Scientific TMT16plex reagent for liver tissues and CAFs with carrier channel samples or with TMT10plex reagent for all the other experiments. 20 μg (for TMT16plex) and 30 μg (for TMT 10Plex) of each sample was labeled with 0.1 mg of TMT reagent dissolved in 50 μL of 100% anhydrous acetonitrile. The reaction was carried out at room temperature for 2 h shaking at 1,000 rpm and quenched adding a 5% hydroxylamine solution. Fully labeled samples were mixed in equal amount and desalted using a 50 mg Sep Pak C18 reverse phase solid-phase extraction cartridge (Waters).

#### High pH peptide fractionation

Desalted TMT-labelled peptides were fractionated using high pH reverse phase chromatography on a C18 column (150 × 2.1 mm i.d. - Kinetex EVO (5 μm, 100 Å)) on an HPLC system (Agilent, LC 1260 Infinity II, Agilent). A two-step gradient was applied, from 1 to 28% B in 42 min, then from 28 to 46% B in 13 min to obtain a total of 21 fractions for MS analysis as previously described.^11^

#### MS analysis on Orbitrap Fusion Lumos and Q-Exactive HF

Fractionated peptides were separated by nanoscale C18 reverse-phase liquid chromatography using an EASY-nLC II 1200 (Thermo Scientific) coupled to an Orbitrap Q-Exactive HF (for the murine kidney cells) or to an Orbitrap Fusion Lumos (all other samples) mass spectrometer (Thermo Scientific). Elution was carried out using a binary gradient with buffer A (water) and B (80% acetonitrile), both containing 0.1% formic acid. Samples were loaded with 6 μL of buffer A into a 50 cm fused silica emitter (New Objective) packed in-house with ReproSil-Pur C18-AQ, 1.9 μm resin (Dr Maisch GmbH). Packed emitter was kept at 50°C by means of a column oven (Sonation) integrated into the nanoelectrospray ion source (Thermo Scientific). Peptides were eluted at a flow rate of 300 nL/min using different gradients which we optimized for three sets of fractions: 1–7, 8–15, and 16–21. Each fraction was acquired for a duration of 190 minutes for the Fusion Lumos or 120 min for the Q-Exactive HF. Eluting peptides were electrosprayed into the mass spectrometer using a nanoelectrospray ion source (Thermo Scientific). An Active Background Ion Reduction Device (ESI Source Solutions) was used to decrease air contaminants signal level. The Xcalibur software (Thermo Scientific) was used for data acquisition. A full scan was acquired at 60,000 resolution at 200 m/z, with a target value of 3e6 ions for a maximum injection time of 20 ms. Higher energy collisional dissociation fragmentation was performed on the 10 most intense ions for the Q-Exactive HF, or during a 3 s cycle time for the Fusion Lumos. Ions were selected within an isolation window of 0.8 m/z, for a maximum injection time of 96 ms, or a target value of 100,000 ions. Peptide fragments were analyzed in the Orbitrap at 60,000 resolution for the Q-Exactive HF, or at 50,000 resolution for the Fusion Lumos. Note that 0.8 m/z isolation window was chosen to mitigate the risk of false positives. Given the 4 Da mass shift introduced by IAA labeling, this window ensures sufficient precursor separation at typical charge states (2+ to 4+), effectively minimizing co-isolation of light and heavy forms. For higher charge states (≥5+), where the isotopic separation approaches 0.8 Da, potential overlap is theoretically possible; however, such species represented only less than 1%, on average, of identified precursors in our dataset and had negligible impact on quantification. Furthermore, the Gaussian transmission profile of the quadrupole reduces ion transmission at the edges of the isolation window, further limiting co-isolation artifacts.

#### MS analysis on Orbitrap Astral

Fractionated peptides (7 fractions) were analyzed with an Orbitrap Astral mass spectrometer (Thermo Scientific) coupled to a Vanquish *Neo* UHPLC system (Thermo Scientific), interfaced with an Easy-Spray source (Thermo Scientific) and equipped with an Aurora XT (75 μm, 25 cm) analytical column (IonOpticks). The column temperature was stabilized at 45°C with a column heater from IonOpticks. 90 min and 12 0min gradients were performed using a binary gradient with buffer A (water, 0.1% formic acid) and B (80% acetonitrile, 0.1% formic acid) and a flow rate of 400 nL/min. Eluent B was ramped from 4% to 31% in 80 min followed by 31%–45% in 10 min for the 90 min gradient and from 4% to 31% in 105 min followed by 31%–45% in 15 min for the 120 min gradient. The Orbitrap Astral MS was operated in DDA mode (positive mode) for all experiments. MS1 spectra were collected in the Orbitrap every 1 s at a resolving power of 120,000 at m/z 200 over m/z 400–1500 with a normalized AGC target of 300% (3e6 charges) and a maximum injection time of 50 ms. MS2 scans were collected in the Astral mass analyzer with an isolation window of 0.5 m/z, normalized collision energy of 38, a scan range of 110–1500 m/z, an AGC target of 100% (1e4 charges), and a maximum injection time of 20 ms.

#### Mass spectrometry raw data analysis

The MS Raw data were processed with MaxQuant software^35^ version 1.6.14.0 and searched with Andromeda search engine,^36^ querying SwissProt^37^ *Homo sapiens* (42,438 entries) or *Mus musculus* (25,198 entries) database. First and main searches were performed with precursor mass tolerances of 20 ppm and 4.5 ppm, respectively, and MS/MS tolerance of 20 ppm. The minimum peptide length was set to six amino acids and specificity for trypsin cleavage was required, allowing up to two missed cleavage sites. MaxQuant was set to quantify on “Reporter ion MS2”, and TMT10 or 16 plex was chose as Isobaric label. Interference between TMT channels was corrected by MaxQuant using the correction factors provided by the manufacturer. The “Filter by PIF” option was activated and a “Reporter ion tolerance” of 0.003 Da was used. Modification by Iodoacetamide heavy or light on Cysteine residues (Carbamidomethylation), Methionine oxidation and protein N-terminal acetylation were allowed as variable modifications. No fixed modification was used in the search. The peptide, protein, and site false discovery rate (FDR) was set to 1%.

### Quantification and statistical analysis

The files from MaxQuant output: “proteinGroups.txt”, “modificationSpecificPeptides.txt”, “peptides.txt” and both heavy and light “Carbamidomethyl (C)Sites.txt” were used for the analysis in Perseus version 1.6.15.0.^38^ The “proteinGroups.txt” file was used for proteome analysis and for normalization of cysteine containing peptides. The modificationSpecificPeptides.txt file was used as main table for peptide quantification. The columns with headers “C count”, “Start/End position”, “Length”, “Leading razor protein” from the peptides.txt file, and the columns with headers “Sequence Features”, “Score diff”, “Localisation prob”, “Position” and “FASTA” from the heavy and light Carbamidomethyl (C)Sites.txt files were merged to the modificationSpecificPeptides.txt file using the corresponding id and “Matching by row” tool in Perseus.

The protein or peptides specified in MaxQuant as “Reverse” and “Potential Contaminants” were removed from all tables. For protein quantification, protein groups indicated as “Only identified by site” and those identified with zero unique peptides were removed from the table. Peptides with “Cys count” lower than 1 were excluded from analysis, and only cysteine containing peptides robustly quantified in all the replicate experiments were normalized to the total protein levels and included in the quantification analysis. “Score difference” and “Localization probability” of the quantified cysteine residues are reported in the Supplementary Data Tables. The annotation of the cysteine residues was based on the sequence features database of UniProt using the Site Annotation built-in tool in Perseus. Gene Ontology annotation analysis was also performed using the built-in tool in Perseus. To assess the significance of regulated cysteine residues between experimental conditions, Student’s t test or ANOVA test were used with a permutation-based FDR below 5%.

## Supplemental information

Table S1. Redox proteome and proteome measured with SICyLIA-TMT of murine fibroblasts treated with H_2_O_2_, related to Figure 1

Table S2. Redox proteome and proteome measured with SICyLIA-TMT of breast cancer-associated fibroblasts treated with H_2_O_2_, related to Figure 2

Table S3. Redox proteome and proteome measured with SICyLIA-TMT with carrier channel to boost heavy IAA signal of breast cancer-associated fibroblasts treated with H_2_O_2_, related to Figure 2

Table S4. Redox proteome and proteome measured with SICyLIA-TMT of murine liver of wild-type (WT) and *Ob/Ob* mice, related to Figure 3

Table S5. Redox proteome and proteome measured with SICyLIA-TMT with carrier channel to boost heavy IAA signal of murine liver of wild-type (WT) and *Ob/Ob* mice, related to Figure 3

Table S6. Redox proteome and proteome measured with SICyLIA-TMT of murine liver of wild-type (WT) and *Ob/Ob* mice run on an Orbitrap Astral, related to Figure 4

Table S7. Costs and time calculation for SICyLIA, SICyLIA-cTMT, and SICyLIA-cTMT run in an Orbitrap Astral, related to Figure 4
